# Effect of Heat Treatment Combined with TG Enzyme Cross-Linking on the Zein–Pea Protein Complex: Physicochemical and Gel Properties

**DOI:** 10.3390/gels10050301

**Published:** 2024-04-28

**Authors:** Yan Li, Chi Wang, Nannan Hu, Yuanhui Zhao, Yuzhu Wu, Jingsheng Liu, Yilin Zhao

**Affiliations:** 1 College of Food Science and Engineering, Ocean University of China, Qingdao 266100, China; qgly1007@163.com (Y.L.); wangchi@163.com (C.W.); zhaoyuanhui@ouc.edu.cn (Y.Z.); 2 Chengde Center for Disease Control and Prevention, Chengde 067000, China; 3 College of Food Science and Engineering, Jilin Agricultural University, Changchun 130118, China; hunannan@163.com (N.H.); wo_shiwuyuzhu@163.com (Y.W.); liujs@163.com (J.L.)

**Keywords:** pea protein, zein, heat treatment, TG enzyme, gel properties

## Abstract

Plant proteins have the advantages of low cost and high yield, but they are still not comparable to animal proteins in processing due to factors such as gelation and solubility. How to enhance the processing performance of plant proteins by simple and green modification means has become a hot research topic nowadays. Based on the above problems, we studied the effect of gel induction on its properties. In this study, a pea protein–zein complex was prepared by the pH cycle method, and the effects of different induced gel methods on the gel properties of the complex protein were studied. The conclusions are as follows: All three gel induction methods can make the complex protein form a gel system, among which the gel strength of heat treatment and the TG enzyme-inducted group is the highest (372.84 g). Through the observation of the gel microstructure, the gel double network structure disappears and the structure becomes denser, which leads to a stronger water-binding state of the gel sample in the collaborative treatment group. In the simulated digestion experiment, heat treatment and enzyme-induced samples showed the best slow-release effect. This study provides a new method for the preparation of multi-vegetable protein gels and lays a theoretical foundation for their application in food processing.

## 1. Introduction

With population growth and healthy diet concepts, the proportion of animal-derived foods in the dietary structure is gradually decreasing, and more and more scholars are focusing on crops and aquatic products [1]. However, plant proteins are usually poorly processed, so how to improve their processing properties and expand their application range has become a hot research topic nowadays. As an important economic crop worldwide, pea has become an important raw material in the food processing industry due to its low price and low allergenicity [2]. Protein is the second most abundant constituent of peas and is a good source of essential amino acids due to its homogeneous amino acid composition [3,4]. During processing, it was found that pea protein has a certain gelation ability, which is beneficial to protect the water and flavor substances in food from being destroyed [5]. Available studies have shown that pea proteins are mainly used as ingredients in food and beverages and as excipients in pharmaceuticals, cosmetics, and composites [6], but the poor solubility of pea proteins compared to soy proteins, which have a wider range of applications, limits their properties such as dispersion, emulsification, and foaming in aqueous systems [7]. Because of solubility and other effects, the gelation of pea protein is also greatly affected [8]. Zein, the main storage protein in corn, was first discovered by Gorham in 1821 and named zein [9]. Due to its unique amino acid composition, zein can only be solubilized in aqueous solutions under specific conditions. According to our preliminary studies [10], zein can dissolve in alkaline solutions and form nanoparticles with pea proteins; whether this would have an effect on the gel properties of the composite proteins needs to be explored further.

Proteins have many excellent properties, such as high nutritional value and good biodegradability, which make them widely used in food and other industries, and they are important delivery systems for active substances [11,12]. Nano-proteins are easy to produce industrially and can be converted into powder, which facilitates storage and transportation. In addition, they can enhance the solubility of hydrophobic actives through higher loading rates, which is more important in the embedding and delivery of functional components [13]. Gelation properties are one of the most important properties of proteins in production and processing. Under the action of external forces, the structure unfolds, and intermolecular cross-linking and aggregation occur. The three-dimensional network structure formed by the equilibrium of intermolecular interaction forces is usually called the gelation of proteins [14]. Gels are usually categorized into single-substance gels and composite gels with multiple similar substances, among which composite gels usually have superior performance as the components in the gel can form functional complementarities, enhancing the gel’s overall performance. Some scholars have studied whey isolate protein [15], a composite system of whey isolate protein and gelatin with good tensile behavior. Wong [16] et al. investigated the pea isolate protein and whey isolate protein system, and the composite gel had the highest gel strength and elasticity at 80% whey isolate protein addition. The gelation of proteins is mainly applied to meat water retention, dairy products [17], and active substance delivery [18].

According to the induced gelation mode, protein gelation can be divided into heat-induced gelation and non-heat-induced gelation. With the gradual deepening of research on the gelation process of proteins, a lot of research results have shown that heat treatment can lead to changes in the structure of protein molecules. During the heating process, protein molecules are thermally denatured and their structure unfolds, exposing a large number of functional sites where interactions can take place, and during the cooling process, protein molecules are entangled and aggregated, forming macromolecular aggregates with a three-dimensional network structure [19]. Non-thermally induced gels mainly include enzyme induction [20], acid-induced gels, and salt-induced gels [21]. Enzymatic treatment is an important, safe, and non-toxic route in the non-thermal-induced gelation process. TG enzymes catalyze the covalent binding between γ-amide groups and ε-amino groups in protein molecules to facilitate the process of protein gelation. Numerous studies have been conducted to show that heat treatment and enzyme treatment play an important role in the protein gelation process, but the effect of the combination of heat treatment and enzyme treatment on the gelation of composite plant protein complexes has not been studied, and there is still no report on whether heat treatment can play a facilitating role in the gelation process of two protein complexes with different solubilities.

Based on previous studies and related literature reports, we proposed the following hypotheses: Can a short heat treatment expand the protein structure and thus improve the effect of TG enzymes? Can zein introduce more amide groups and enhance the action of TG enzymes? In this study, the interaction between pea isolate protein and corn alcohol-soluble protein was carried out to simulate the process of protein gel formation and to explore the interaction mechanism between the two proteins in solution as well as in the gel network structure. This provides a theoretical basis for the interaction of composite plant protein systems and improves the performance of composite proteins and processing applications.

## 2. Results and Discussion

### 2.1. Effects of Different Treatments on Physicochemical Properties of Complex Protein Solutions

#### 2.1.1. Solubility

**Figure 1:**
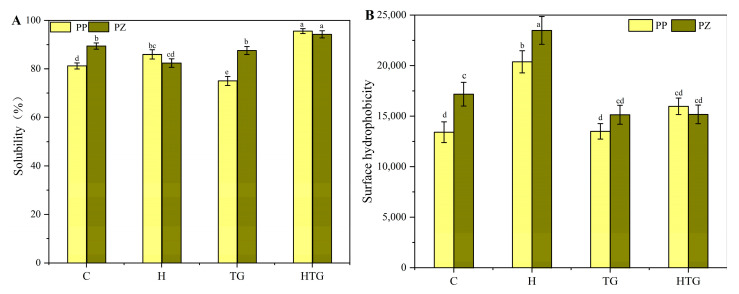
Effects of different induction modes on protein solubility (**A**) and surface hydrophobicity (**B**). a–e Means in the same group with different letters were significantly different (*p* < 0.05) from each other.

Solubility is one of the most intuitive factors in the ability of proteins to form gels and in influencing gel properties. The determination of the solubility of protein samples with different induction methods can help to explain the reasons for the changes in the gel properties of complex proteins. The effect of different induction methods on protein solubility is shown in Figure 1A, where C is the sample without any treatment and H, TG, and HTG are the heat treatment, TG enzyme treatment, and co-treatment group samples. The significant decrease in the solubility of the composite proteins during heat treatment may be attributed to the fact that the introduction of zein increased the area of hydrophobic regions after the unfolding of the composite protein structure. And the soluble aggregates produced by dissociation were not sufficient to counteract the effect of the exposure of the hydrophobic regions on the solubility. The solubility of pea proteins induced by TGase was significantly reduced by the TGase-induced acyltransfer reaction of inter- or intra-protein molecules with amide groups, which resulted in the cross-linking of protein molecules with each other to form aggregates and the precipitation of these aggregates due to the excessively large molecular weight and other reasons, leading to a significant decrease in solubility [22,23]. While the solubility of all the samples that underwent heat treatment combined with the TG enzyme cross-linking reaction increased, which may be due to protein molecular chain breakage caused during the heat treatment process, the resulting peptides participated in the TG enzyme cross-linking reaction and formed aggregates with molecular weights smaller than those of the TG enzyme cross-linking samples alone, which played a contributing role in the increase in solubility.

#### 2.1.2. Surface Hydrophobicity

Figure 1B shows the effects of different induction methods on the surface hydrophobicity of proteins. The composite protein samples showed a significant increase in surface hydrophobicity after heat treatment, which was attributed to the large number of hydrophobic groups carried by zein. In the group of TGase-treated samples, the proteins showed a decrease in surface hydrophobicity, probably due to the large number of hydrophobic groups carried by zein, which, during the action of Tgase, altered some of the amino acid residues of the proteins, undergoing acyltransfer reactions and forming covalent bonds between the molecules to maintain the structure, which in turn had an effect on the surface hydrophobicity of the proteins [24]. In the preheated synergistic Tgase-treated group, none of the hydrophobicity of the surface of the composite proteins changed significantly, probably due to the fact that the hydrophobic groups introduced by the maize alkyd proteins in this protein ratio undergo complete cross-linking reactions under the action of Tgase, and the increase in their solubility may be related to this, which increases the interactions between the protein molecules in the solution and positively affects the formation of the structure of the gel network.

### 2.2. Effects of Different Treatments on the Properties of Composite Protein Gels

#### 2.2.1. Textural Properties

**Figure 2:**
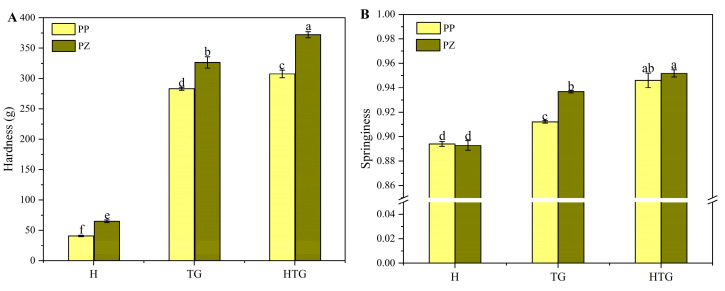
Changes in gel hardness (**A**) and springiness (**B**) of different induction methods. a–f Means in the same group with different letters were significantly different (*p* < 0.05) from each other.

The effects of thermal induction, enzyme induction, and synergistic enzyme cross-linking by preheating treatment on the textural properties of the gels are shown in Figure 2. Because untreated protein solutions do not form gels, this part of the study does not include samples from the untreated group. The gel formed by thermal induction alone had highly significant differences compared to the remaining two modes of induction, and the gel structure was looser and not suitable for processing in the food industry. Enzyme-induced gels showed a significant increase in gel strength compared to heat-induced gels. Preheating before TG enzyme cross-linking also had a positive effect on the gel strength of the samples. Enzyme treatment can lead to the formation of covalent binding between protein molecules and the establishment of a stronger gel network structure [25]. Preheating treatment before the cross-linking action of Tgase also has a certain enhancement effect on the gel strength of the samples and can unfold the folded structure of the proteins to provide more sites for Tgase to act, resulting in faster gel formation and a denser structure.

#### 2.2.2. Water Retention and Moisture Distribution

**Figure 3:**
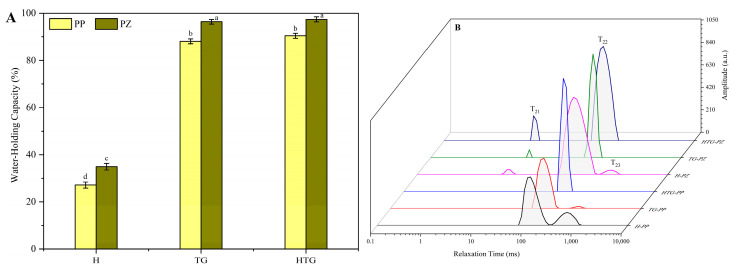
The water-holding capacity (**A**) and water distribution (**B**) of the gels with different induction methods. a–d Means in the same group with different letters were significantly different (*p* < 0.05) from each other.

The way in which gels form directly affects the construction of the gel network structure, and the change in the gel network structure will also have a greater impact on the water-holding property and water distribution. The water-holding capacity and water distribution were determined for three different gel-inducing modes, and the results are shown in Figure 3A,B.

Figure 3A shows the results of the water-holding capacity of different gel samples. According to the results of previous studies, the network structure formed by thermal induction was loose and easy to collapse and destroy by external forces, which made the water molecules in the network structure easy to remove. The gel structure formed after TG enzyme induction was more compact, and the gel water-holding capacity was significantly improved. Combined with the characterization of the moisture distribution of each sample in Figure 3B, it can also be seen that the absorption peaks corresponding to free water in the gels were reduced after TG enzyme induction, and the free-water peaks disappeared from the TGPZ and HTGPZ samples, so that the water-binding capacity was improved. The water-holding property of the samples with preheating treatment in combination with enzyme-induced gel formation was not significantly changed, but the position of their water distribution peaks appeared to be shifted to the left, and the water molecules were more firmly bound.

#### 2.2.3. Microscopic Morphology

**Figure 4:**
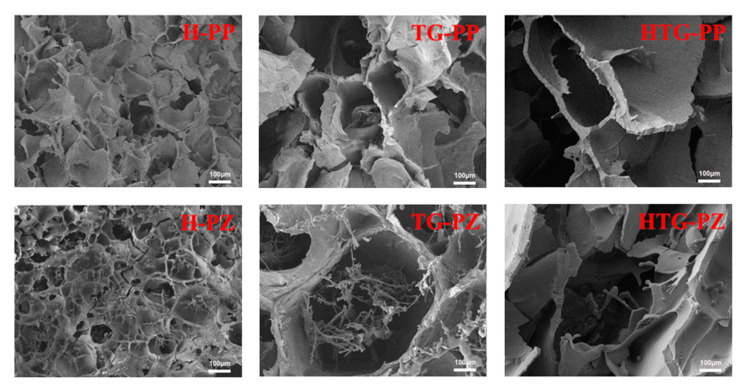
Micromorphology of gels.

The microscopic morphology can intuitively respond to the structural changes in the gel and provide a reference for understanding the mechanism of changing gel properties. We used an electron microscope to observe the microscopic morphology of the gels, as shown in Figure 4.

In previous studies, it was found that the gel samples with the addition of zein were affected by the change in gel structure according to the addition ratio, whereas the gel samples with a mass ratio of 8:2 showed optimal gel properties. A comparison of the samples with different gelation methods revealed that finer fragmented filamentous structures of zein were observed in the gel network in all samples. In the heat-induced gel, the dual-network structure was more obvious, but the two-layer network was not combined, which played a destructive role in the overall network structure of the gel; in the gel samples induced by TG enzyme, the zein network was flooded in the network structure of the main body of the pea protein, which played a certain role in supporting the gel structure, and the gel structure was more dense, which corresponded to the results shown by the water-holding property and the texture and structure properties; in the gel samples cross-linked by heat treatment synergized with TG enzyme, it was clearly observed that zein structure was not visible in the gel network. In the heat treatment synergistic TG enzyme cross-linking gel samples, it can be clearly observed that the network formed by corn alcohol-soluble proteins and pea proteins has been combined to jointly construct the main gel network structure, which presents a denser and stronger phenomenon and also confirms the reason for the change in gel properties from the microstructure.

### 2.3. Effects of Different Treatments on the Effect of Composite Protein Gels Loaded with Curcumin

Based on our previous findings [10], composite proteins with curcumin-loading capacity and proteins with a stable three-dimensional network structure after gel formation may enhance the protection of functional actives. Therefore, the loading capacity of gel samples with different induction methods was determined, and the results are shown in Table 1. The loading efficiency and loading capacity of the composite protein particles were 65.23% and 6.52% in our previous study. The thermal induction mode resulted in a decrease in the loading efficiency and loading capacity of the gels. This may be due to the fact that during heat-induced gelation, the composite protein particles experienced a decrease in curcumin content due to the unfolding of the structure by heat, leading to a decrease in the loading efficiency and loading capacity of the gel. The increase in loading efficiency and loading capacity of TG enzyme-induced gel samples may be due to the fact that the enzyme-induced gel network structure is formed more rapidly and curcumin has very low solubility in water, so the rapid formation of the network structure is beneficial for the loading of curcumin, which is uniformly wrapped in the gel network structure, increasing the loading efficiency and loading capacity of the sample gels. The loading efficiency and loading capacity of the preheated synergistic TG enzyme-induced samples were further improved. After the preheating treatment, the unfolding degree of the protein molecules increased, the network structure was formed more rapidly and densely, and the curcumin molecules were dispersed more uniformly in the gel network with a larger loading capacity.

### 2.4. Release Properties of Gels

**Figure 5:**
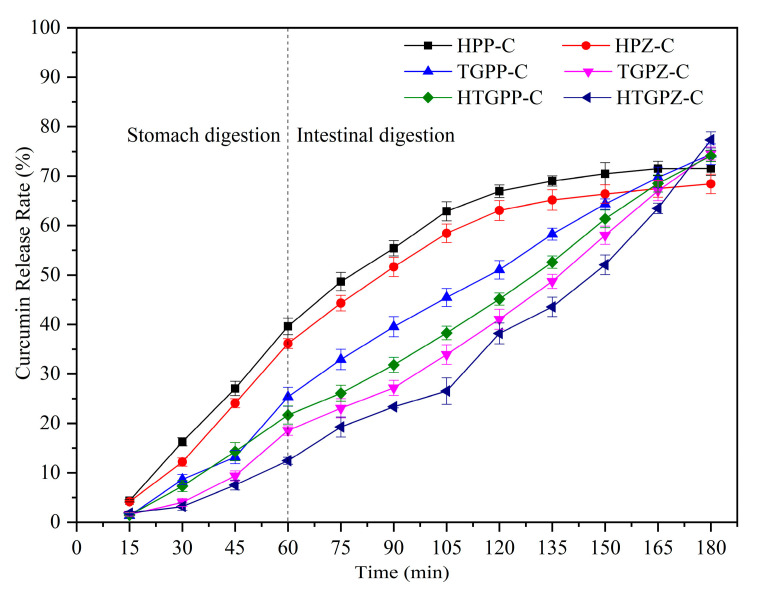
Curcumin release rates of gels in the stomach and intestines with different induction methods.

Gels are often applied as carriers due to their unique network structure, good stability, and protection of functionally active substances. Therefore, the digestive release behavior of gel carriers in the gastric and intestinal tracts is crucial. The release behavior of gels in the gastric and intestinal tracts with different induction methods is shown in Figure 5 and Table 2.

The release behavior of the gels was divided into two stages: gastric and intestinal. At the beginning of digestion, the release rate of heat-induced gel samples was higher than that of the rest of the gel samples; at the end of gastric digestion, the release rate of curcumin from the gel samples differed significantly, and the release of the composite protein gel induced by the preheated synergistic TG enzyme was the least, which had the same pattern with its structural density. The dense gel network structure is more resistant to gastric juice and pepsin, which can protect the active substances encapsulated in it more effectively. After simulated intestinal digestion, the sample release rate of the heat-induced gel gradually decreased because the curcumin content measured in this section was the accumulation of curcumin from the beginning of digestion to the moment of sampling, and the accumulation rate of curcumin in the digested liquid showed a decreasing trend. The heat-induced gel did not play an obvious role in slowing down the release of curcumin, and the curcumin that was rapidly released in the process of digestion may be at risk of decomposition and inactivation; the TG enzyme-induced gel basically maintained the same release rate and continued to release during the whole digestion process, and its protective effect on the active substances was better than that of the heat-induced gel; the preheating treatment synergized with the TG enzyme-induced gel showed the phenomenon of gradually increasing curcumin release during the whole digestion process. At the early stage of digestion, the release of curcumin was relatively slow, and after intestinal digestion, the gel network structure was destroyed, and curcumin was rapidly released. This facilitated the active substances reaching the small intestine and being absorbed by the human body, which played a role in slow release. The results showed that the protective effect of the gel carriers on the active substances was closely related to the degree of structural compactness, and the dense gel network could help to enhance the protective effect of the gel carriers on the active substances.

## 3. Conclusions

The effects of different gel induction methods on the gel properties of the pea protein–zein complex were studied. The preheating treatment combined with TG enzyme cross-linking induced the sample to have the maximum gel strength, and the water retention improved by about 1.78 times compared with heat-induced gel. The morphology of the gel was observed, and the gel networks of the two proteins combined with each other, resulting in a closer structure. The gel prepared by this method has a good slow-release effect. This study provides a new research idea for the preparation of various plant protein composite gels and lays a theoretical foundation for the application of plant protein gels.

## 4. Materials and Methods

### 4.1. Materials

Pea protein (Pp) (90.47% protein, *w*/*w*) was provided by Xi’an Zelong Biological Technology Co. Ltd. (Xi’an, China). Zein (92.6% protein, *w*/*w*) was obtained from Ryon Biological Technology Co. Ltd. (Shanghai, China). The protein content was determined by the Kjeldahl method. Potassium bromide (KBr) was purchased from Sigma Aldrich Co. Potassium bromide (KBr) was purchased from Sigma Aldrich Co. (St. Louis, MO, USA). TG was obtained from the Hon-grunbaoshun Technology Company (Beijing, China). All other chemicals used in this work were of analytical grade.

### 4.2. Preparation of Pea Isolate Protein–Corn Alcohol-Soluble Protein Complex Stock Solution

Based on previous studies [10], a composite protein solution with a protein content of 15% was prepared by a pH-driven method, in which the ratio of pea protein to zein was 8:2. Pure pea protein was used as a control, and the treatment process was consistent with the sample. Briefly, 12 g of pea protein was dispersed in 100 mL of distilled water, the pH was adjusted to 12 with 2 M NaOH after magnetic stirring, 3 g of corn alkyd soluble protein was added, magnetic stirring was performed to make it fully dissolved, and the pH of the solution was adjusted to 7 using 2 M HCl. The resulting solution was the composite protein stock solution. The relevant properties of the composite protein were explained in detail in our previous study [10].

### 4.3. Protein Solution Preparation for Different Treatments

Heat induction: 1% protein solution was heated in a 90 °C water bath for 30 min (named H); enzyme treatment: 1% solution was mixed with TG enzyme at 20 U/g enzyme addition and held at 45 °C for 1 h (named TG); co-treatment: 1% protein solution was heated at 90 °C for 3 min, mixed with TG enzyme, and held at 45 °C for 1 h (named HTG).

### 4.4. Physicochemical Properties of Complex Proteins

#### 4.4.1. Solubility

Referring to the method of Lowry et al. [26], the protein solution was centrifuged at 4000× *g* for 10 min, the supernatant was taken, and the protein content in the supernatant was determined using a protein content kit to obtain the protein mass in the supernatant, m_1_, and the amount of protein added, m_2_. The protein solubility was calculated by the following equation:

(1) $$Protein\;solubility\;(\%)=\frac{m_{1}}{m_{2}}\times 100$$

#### 4.4.2. Surface Hydrophobicity

The ANS probe was selected to label the hydrophobic groups on the surface of the protein, and the sample solution was diluted to 0.001 mg/mL (from 1 mg/mL). A total of 2 mL of the sample dilution was accurately pipetted to add 20 μL of ANS, added to a 96-well plate, and incubated at 25 °C for 3 min. The fluorescence intensity was measured immediately by using an enzyme marker with an excitation wavelength of 390 nm and an emission wavelength of 470 nm, and a curve was plotted using fluorescence intensity as the vertical coordinate and protein concentration as the horizontal coordinate. The initial slope is the surface hydrophobicity of the sample [27,28].

### 4.5. Composite Protein Gel Preparation with Different Induction Modes

Thermal induction: the protein solution was heated in a 90 °C water bath for 30 min, cooled with ice water, and stored in a refrigerator at 4 °C overnight to fully mature the gel (named H-PZ); enzyme induction: TG enzyme (20 U/g) was added to the composite protein reserved solution and held at 45 °C for 1 h to form the gel (named TG-PZ); co-treatment group: the reserved solution was first heated in a 90 °C water bath for 3 min, cooled, and then added to the TG enzyme (20 U/g) and held at 45 °C for 1 h to form the gel (named HTG-PZ).

### 4.6. Gel Performance Test

#### 4.6.1. Textural Properties

The gel samples were subjected to the determination of their textural properties by means of a TA-XT Plus texturometer. The probe model P-0.5, selected with reference to Salvador et al. [29], was improved with a test and post-test rate of 1 mm/s, a probe downward pressure distance of 10 mm, and a trigger force of 1 g. The first peak of the curve in the test results was recorded as the point of gel rupture of the samples, i.e., the gel strength.

#### 4.6.2. Water Retention and Moisture Distribution

Referring to Campbell [30], the method of Campbell et al. was slightly improved by leaving the gel sample at room temperature for 30 min to make the temperature of the gel consistent with that of the room temperature, draining the moisture from the surface of the sample to exclude the effect of condensation on the test, cutting 5 g of the sample and placing it in a centrifuge tube, and centrifuging it at 4 °C for 10 min at 1000× *g*. The sample was then dried to remove the water discharged from the surface of the sample, and then centrifuged for 10 min at 4 °C. The water discharged from the surface of the sample was sucked up, and the weight of the sample before and after centrifugation and the weight of liquid discharged were recorded. The weight of the sample before and after centrifugation and the weight of liquid discharged were recorded and calculated according to the following formula:

(2) $$Water\;Holding\;Properties\;(\%)=\frac{Gel\;weight-Weight\;of\;discharged\;water}{Gel\;weight}\times 100$$

With reference to Li et al.’s method [31], a 5 g gel sample was placed in a 15 mm NMR tube and subjected to low-field strength NMR determination. The measurement temperature was 20 °C, and the scan was repeated four times. The moisture relaxation time spectra of the samples were obtained, and the integrals were calculated, corresponding to A_21_, A_22_, and A_23_, which corresponded to the percentage of the three different states of moisture in the total moisture content. T_21_, T_22_, and T_23_ were the relaxation times of bound water, semi-bound water, and free water, respectively.

#### 4.6.3. Microscopic Morphology

The microstructure of the samples was observed using SEM electron microscopy according to the method reported by Zhao et al. [32]. Liquid samples were frozen at −80 °C and fixed using conductive adhesive. The samples were sprayed with gold and then observed under an electron microscope at an accelerating voltage of 10 kV for magnification.

### 4.7. Preparation of Curcumin-Loaded Gels

According to the method of our previous study [10], 12 g of pea protein was added to 100 mL of distilled water with magnetic stirring for 30 min, and the pH was adjusted to 12 using 2 M NaOH. A total of 3 g of maize alkyd proteins were added, and curcumin was added after solubilizing. The pH was adjusted to 2 M HCl. The gel was prepared according to the method described previously. The amount of curcumin added was 10% of the total protein content.

### 4.8. Load Efficiency and Load Capacity of Gels

According to the method of Zhao et al. [33], 0.5 g of freeze-dried gel samples was accurately weighed and placed in a centrifuge tube, and a certain amount of 80% ethanol was added to make the protein concentration 1 mg/mL, vortexed and shaken so that curcumin was dissolved in 80% ethanol, and then centrifuged at 4000× *g* for 15 min. The absorbance was measured at 426 nm, and the curcumin content was calculated from the standard curve of curcumin in 80% ethanol. The loading efficiency and loading capacity were calculated using the following equation:

(3) $$Load\;Efficiency\left(\%\right)=\frac{Curcumin\;content\;in\;gel\left(mg\right)}{Added\;curcumin\;content\left(mg\right)}\times 100$$

(4) $$Load\;Capacity\left(\%\right)=\frac{Curcumin\;content\;in\;gel\left(mg\right)}{protein\;content\left(mg\right)}\times 100$$

### 4.9. Digestive Release of Composite Gels

We slightly modified the method of Jiang et al. [34] to simulate digestion in vitro. Briefly, 2 mL of 4 mg/mL pepsin solution (dissolved in 0.1 M HCl) was added to 30 mL of sample. The pH of the mixed solution was adjusted to 2.0 and incubated for 1 h at 37 °C under continuous shaking (100 rpm). Gastric digestion was terminated by adjusting the pH to 5.3 using 0.9 M NaHCO_3_. The gastric digest was mixed with 9 mL of trypsin (2 g/L) as well as bile extract (12 mg/mL). Trypsin and bile extract were dissolved in 0.1 M NaHCO_3_, and the pH was adjusted to 7.5 to simulate intestinal digestion. The samples were incubated for 2 h at the same temperature and oscillation conditions. The curcumin content in the digest was analyzed by measuring the absorbance at 426 nm according to the method described previously (standard curve method). The ratio of curcumin content in the digest to the initial curcumin content was used to quantify the curcumin release behavior.

### 4.10. Statistical Analysis

All samples were prepared in triplicate unless otherwise specified. The data were shown as the average of triplicate measurements and analyzed by one-way analysis of variance (ANOVA) using SPSS 22.0 for Windows software (SPSS Inc., Chicago, IL, USA), and significant differences in means (*p* < 0.05) were determined using the Duncan’s multiple range test.

## Figures and Tables

**Table 1:** Gel loading efficiency and loading capacity of different induction methods.

	H-PP-C	H-PZ-C	TG-PP-C	TG-PZ-C	HTG-PP-C	HTG-PZ-C
LE (%)	79.63 ± 1.17 ^b^	65.12 ± 1.46 ^c^	85.69 ± 1.97 ^a^	84.27 ± 1.45 ^a^	86.32 ± 1.16 ^a^	85.92 ± 1.77 ^a^
LC (%)	7.96 ± 0.12 ^b^	6.51 ± 0.15 ^c^	8.57 ± 0.20 ^a^	8.43 ± 0.15 ^a^	8.63 ± 0.12 ^a^	8.59 ± 0.18 ^a^

Different letters in the graph represent significant differences (*p* < 0.05).

**Table 2:** Curcumin release rates of gels in the stomach and intestines with different induction methods.

	15	30	45	60	75	90	105	120	135	150	165	180
H-PP-C	4.35 ^a^	16.26 ^a^	27.05 ^a^	39.62 ^a^	48.67 ^a^	55.41 ^a^	62.89 ^a^	66.94 ^a^	69.02 ^a^	70.48 ^a^	71.53 ^a^	71.56 ^c^
H-PZ-C	4.17 ^a^	12.18 ^b^	24.07 ^b^	36.14 ^b^	44.31 ^b^	51.63 ^b^	58.44 ^b^	63.06 ^b^	65.18 ^b^	66.39 ^b^	67.47 ^c^	68.43 ^d^
TG-PP-C	1.32 ^b^	8.66 ^c^	13.18 ^c^	25.37 ^c^	32.92 ^c^	39.55 ^c^	45.47 ^c^	51.06 ^c^	58.27 ^c^	64.35 ^bc^	69.73 ^bc^	74.42 ^bc^
TG-PZ-C	1.58 ^b^	4.02 ^d^	9.39 ^d^	18.56 ^e^	23.06 ^d^	27.17 ^e^	33.91 ^e^	41.05 ^e^	48.73 ^e^	58.04 ^c^	67.04 ^c^	74.58 ^bc^
HTG-PP-C	1.42 ^b^	7.31 ^c^	14.28 ^c^	21.69 ^d^	26.09 ^d^	31.83 ^d^	38.27 ^d^	45.14 ^d^	52.61 ^d^	61.37 ^c^	68.52 ^bc^	74.09 ^c^
HTG-PZ-C	1.86 ^b^	3.17 ^d^	7.52 ^d^	12.43 ^f^	19.26 ^e^	23.33 ^f^	26.53 ^f^	38.19 ^e^	43.55 ^f^	52.07 ^d^	63.49 ^d^	77.33 ^a^

Different letters in the graph represent significant differences (*p* < 0.05).

## Data Availability

The raw data supporting the conclusions of this article will be made available by the authors on request.
